# Challenges and Opportunities for Emergency Department Sepsis Screening at Triage

**DOI:** 10.1038/s41598-018-29427-1

**Published:** 2018-07-23

**Authors:** Michael R. Filbin, Jill E. Thorsen, James Lynch, Trent D. Gillingham, Corey L. Pasakarnis, Roberta Capp, Nathan I. Shapiro, Theodore Mooncai, Peter C. Hou, Thomas Heldt, Andrew T. Reisner

**Affiliations:** 1 Department of Emergency Medicine, Massachusetts General Hospital, 55 Fruit Street, Boston, MA 02114 United States; 2 Department of Electrical and Biomedical Engineering, Massachusetts Institute of Technology, 45 Carleton Street, E25-330, Cambridge, MA 02139 United States; 3 Department of Emergency Medicine, University of Colorado Anschutz Medical Campus, Leprino Building, 12401 East 17th Avenue, Aurora, CO 80045 United States; 4 Department of Emergency Medicine, Beth Israel Deaconess Center, 330 Brookline Avenue, Boston, MA 02215 United States; 5 Department of Emergency Medicine, Brigham and Women’s Hospital, 75 Francis Street, Boston, MA 02115 United States

## Abstract

Feasibility of ED triage sepsis screening, before diagnostic testing has been performed, has not been established. In a retrospective, outcome-blinded chart review of a one-year cohort of ED adult septic shock patients (“derivation cohort”) and three additional, non-consecutive months of all adult ED visits (“validation cohort”), we evaluated the qSOFA score, the Shock Precautions on Triage (SPoT) vital-signs criterion, and a triage concern-for-infection (tCFI) criterion based on risk factors and symptoms, to screen for sepsis. There were 19,670 ED patients in the validation cohort; 50 developed ED septic shock, of whom 60% presented without triage hypotension, and 56% presented with non-specific symptoms. The tCFI criterion improved specificity without substantial reduction of sensitivity. At triage, sepsis screens (positive qSOFA vital-signs and tCFI, or positive SPoT vital-signs and tCFI) were 28% (95% CI: 16–43%) and 56% (95% CI: 41–70%) sensitive, respectively, p < 0.01. By the conclusion of the ED stay, sensitivities were 80% (95% CI: 66–90%) and 90% (95% CI: 78–97%), p > 0.05, and specificities were 97% (95% CI: 96–97%) and 95% (95% CI: 95–96%), p < 0.001. ED patients who developed septic shock requiring vasopressors often presented normotensive with non-specific complaints, necessitating a low threshold for clinical concern-for-infection at triage.

## Introduction

Septic shock has a substantial mortality rate, regardless of the specific resuscitation strategy employed^1–3^. A leading opportunity to improve patient outcomes may be to reduce delays in therapy, and to initiate therapy earlier than today’s standard-of-care, for mitigation or prevention of critical illness. Early identification could reduce the time to first antibiotic which, according to one recent meta-analysis of early goal directed therapy (EGDT) trials, explained 96 to 99% of the entire survival differences between EGDT and controls^4^. New Centers for Medicare and Medicaid Services metrics are focused on early sepsis care, essentially mandating reliable and early sepsis identification and early initiation of diagnostic testing and treatments^5^.

Since July 2013, the Emergency Department (ED) in our large, urban tertiary care hospital has used the Shock Precautions on Triage (SPoT) Sepsis rule^6^, intended to help ED clinicians identify potentially septic patients prior to diagnostic testing, upon triage or shortly thereafter. The SPoT Sepsis rule consists of a vital-signs criterion, based on systolic blood pressure (SBP) and Shock Index (SI, equal to heart rate (HR)/SBP) in conjunction with a triage concern-for-infection (tCFI) criterion. The tCFI criterion is based on the patient’s presenting complaints and past medical history; it does not require diagnostic testing.

In 2016, the international Sepsis Definitions Task Force advocated for the use of the quick Sepsis-related Organ Failure Assessment (qSOFA) score for sepsis screening based on three vital signs: SBP, respiratory rate (RR), and Glasgow Coma Scale (GCS). The authors advised that a positive qSOFA score should “prompt consideration of *possible* [emphasis added] infection in patients not previously recognized as infected”^7^. Subsequent analyses validated the mortality prediction of qSOFA in ED patients ultimately treated for infection^8,9^, while other reports questioned its sensitivity for sepsis^10^ and whether qSOFA was suitable for detecting sepsis during the initial hours of ED care^11^.

Two questions naturally arise in considering how to detect ED patients with sepsis as early as possible. First, what is the role of vital signs, either in the SPoT Sepsis rule or qSOFA score? In reporting favorable test characteristics for qSOFA, Singer *et al*. examined only the most abnormal vital signs documented throughout the ED stay^7^. However, if the goal is early identification of sepsis, it is important to consider temporal behavior of vital signs, and determine what vital-sign abnormalities can be detected *earliest*: upon triage, or shortly thereafter.

Second, it is important to clarify how clinicians should determine “possible infection in patients not previously recognized as infected”^7^. This essential question may be challenging upon ED arrival, and has not yet been sufficiently addressed, particularly prior to diagnostic testing in patients presenting to the ED. Accurate determination of “possible infection” is pivotal to the usefulness of sepsis screening. Failure to identify those *with* infection reduces sensitivity and negates the value of screening. Failure to filter out patients *without* infection will lead to excess false-positives which also reduces the operational value of screening. The SPoT Sepsis rule does include guidelines for determining possible infection, while the Sepsis Definitions Task Force recommendations do not explicitly specify how to identify possible infection.

To answer these basic questions about the feasibility of sepsis screening upon ED patient triage, we evaluated the SPoT and qSOFA vital-signs criteria to investigate their suitability for ED sepsis screening. Also, we evaluated the tCFI criterion of the SPoT Sepsis rule to examine whether it can help identify which patients who meet the vital-signs criteria do indeed have possible infection.

## Methods

### Design and setting

This was a retrospective observational study from a single urban ED, approved by Partners Human Research Committee (PHRC) Institutional Review Board (IRB), and all methods were performed in accordance with the relevant guidelines and regulations. A waiver of informed consent was granted by the IRB.

### Patients and Outcome

Our primary outcome was septic shock in the ED, defined as initiation of vasopressor infusion in the ED and direct admission from the ED to an Intensive Care Unit (ICU) with antibiotics initiated in 24 hours or less for the documented indication of suspected sepsis. The secondary outcome was admission to an ICU within 24 hours with antibiotics initiated for the documented indication of suspected sepsis.

The SPoT Sepsis rule was previously derived in adult ED patients with septic shock from one calendar year, 2009 (“derivation cohort”)^6^. For the current analysis, we re-evaluated this cohort to better describe the clinical characteristics of patients with ED septic shock and the sensitivity of the sepsis screening rules. This cohort was originally identified via electronic query of the hospital billing system, selecting all patients with *International Classification of Diseases*, 9^*th*^ *Revision* hospital discharge diagnosis (ICD-9) codes for sepsis syndromes (995.90, 995.91, 995.92, 785.52), or ICD-9 codes for infection and organ dysfunction as described by Angus^12^. Patients who met those ICD-9 screening criteria underwent a physician chart review to confirm the presence of symptoms ascribable to sepsis while in the ED (to filter out patients who developed sepsis after being admitted to the hospital for other illnesses), and to confirm subsequent initiation of vasopressor therapy in the ED and admission to an ICU with antibiotics initiated in 24 hours or less for the documented indication of suspected sepsis.

We also examined a *de novo* cohort consisting of all adult ED patients, with and without septic shock, from three different 1-month intervals in a subsequent calendar year (“validation cohort”), to evaluate the full operating characteristics of the sepsis screening rules. This number of months (i.e., three months) was determined by a power calculation: 90% power to detect different sensitivities at triage, assuming comparable incidences of sepsis and comparable sensitivities from the derivation cohort. Specifically, we randomly selected a 1-month interval. To mitigate seasonal bias, we also selected two additional 1-month intervals four months prior and four months after the initial 1-month period. For the validation cohort, all adult ED visits were identified (using our ED informatics system) with subsequent chart review of patients who were admitted to an ICU (either directly or within 24 hrs of ED presentation) to determine the presence of symptoms ascribable to sepsis while in the ED, initiation of vasopressor therapy in the ED, and antibiotics initiated in 24 hours or less for the documented indication of suspected sepsis. Records were reviewed by two independent reviewers, with discrepancies resolved by re-inspection of the chart.

### Measurements

Below, we describe (a) the SPoT Sepsis rule; (b) our methodology for applying the SPoT Sepsis rule retrospectively to the derivation and validation cohorts; and (c) our methodology for applying the qSOFA score retrospectively to the cohorts.

**Figure 1:**
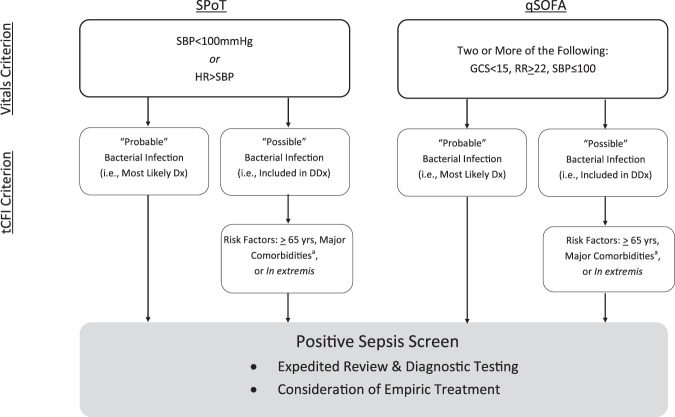
Schematic of investigational sepsis screening tools. Dx = diagnosis; DDx = differential diagnosis. ^a^Major comorbidities: immunosuppressive therapy; active cancer; long-term institutionalization; permanent neurologic disability; chronic dialysis; congestive heart failure limiting daily function; chronic pulmonary disease with oxygen–dependence; liver disease with evidence of cirrhosis; presence of percutaneous tubes or drains; prior sepsis; or recent major surgery.

The SPoT Sepsis rule, summarized in Fig. 1, consists of a vital-signs criterion and a triage tCFI criterion^6^. The vital-signs criterion is positive if either SBP < 100 mmHg and/or SI > 1 (i.e., HR > SBP). The tCFI criterion is either positive or negative, as a function of the clinician’s judgment of whether bacterial infection is “possible” or “probable” and of the patient’s risk factors. “Possible” bacterial infection means that the infection is on the clinician’s differential diagnosis based on the patient’s clinical presentation, along with other possible diagnoses. “Probable” bacterial infection means that bacterial infection is judged to be *more likely than any other possible diagnosis*. The tCFI criterion is met anytime there is “probable” bacterial infection, or when there is “possible” bacterial infection in patients with a sepsis risk factor. A Sepsis Risk Factor was considered one of the following: age ≥65 years; immunosuppressive therapy; active cancer; long-term institutionalization; permanent neurologic disability; chronic dialysis; congestive heart failure limiting daily function; chronic pulmonary disease with oxygen–dependence; liver disease with evidence of cirrhosis; presence of percutaneous tubes or drains; prior sepsis; recent major surgery; or any patient *in extremis*. In our actual clinical implementation of the SPoT Sepsis rule, clinicians are advised to re-evaluate the tCFI criterion whenever diagnostic data become available, e.g., upgrade from “possible” to “probable” after identifying a pneumonia by chest x-ray.

To determine whether or not patients met the SPoT Sepsis rule based on ED documentation, we conducted a retrospective chart review as follows (note, this chart review was conducted independently of the chart review for identifying the outcome). In the first step, we evaluated documented vital signs for each patient to determine the earliest time that the SPoT Sepsis vital-signs criterion was met, if at all. For the derivation cohort, this entailed paper chart review by two independent reviewers, with discrepancies resolved by re-inspection of the chart. For the validation cohort, whose clinical documentation was available electronically, this entailed an electronic database query, with 50 cases randomly selected to validate that the query was accurate. In the second step, for patients who met the SPoT Sepsis vital-signs criterion, two independent reviewers examined the patients’ charts to determine whether the tCFI criterion was met. The documented Assessment and Plan from the patient’s ED encounter, as well as the initial ED orders, were reviewed to determine retrospectively whether infection was judged at least “possible” by the treating clinicians. By protocol, reviewers were not to consider data from later in the patient’s ED stay, and were blinded to the patient’s post-ED course. Explicit documentation that stated that infection was the most likely diagnosis prior to diagnostic testing results, or initiation of antibiotics prior to diagnostic testing results, were taken as indicators that infection was deemed “probable” by the clinicians. Explicit documentation that infection was on the differential diagnosis, or ordering diagnostic tests for infection upon the initial provider evaluation, were taken as indicators that infection was deemed “possible” by the clinicians. The presence or absence of sepsis risk factors was determined exclusively using ED documentation (i.e., not relying on documentation unavailable to ED caregivers). For the minority of cases when tCFI could not be determined from the ED documentation, we performed a *de novo* determination of the likelihood of infection. To be classified as “probable bacterial infection” patients required documented systemic symptoms (e.g., fever or malaise), localizing signs and/or symptoms indicating the locus of infection (e.g., productive cough or flank pain), and an absence of any signs and/or symptoms suggesting alternative diagnoses (e.g., no rhinorrhea, no radiation into the left arm). For calibration purposes, 67% of the charts were reviewed independently by two team members, and disagreements were adjudicated by the consensus of three team members. Cohen’s Kappa was computed to evaluate the inter-rater reliability.

**Figure 2:**
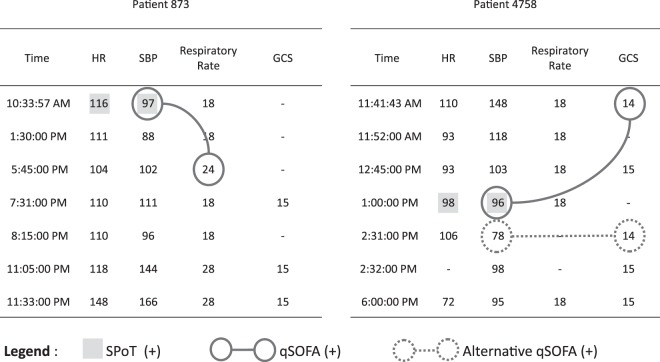
Case examples applying SPoT and qSOFA vital-signs criteria. The SPoT Vital-Signs Criterion was positive when SBP < 100 mmHg and/or HR > SBP; the earliest time that the criterion was met is indicated by shaded boxes in the two case illustrations. The qSOFA Vital-Signs Criterion was positive at the earliest time that at least two of the three constituent conditions were met: (i) Respiratory rate ≥ 22 per min; (ii) GCS < 15; and (iii) SBP ≤ 100 mmHg.7 At any time that there was one documented vital-sign abnormality, we checked earlier in the record to see if there was prior documentation of a second vital-sign abnormality that would make the qSOFA vital-signs criterion positive; the earliest time that the criterion was met is indicated by the later of the connected solid circles in the case illustrations. The Alternative qSOFA Vital-Signs Criterion was also explored, which required at least two of the three constituent qSOFA conditions be met within the same set of documented vital signs; the earliest time that the Alternative qSOFA criterion was met is indicated by dashed circles in right-hand panel, whereas this criterion was never met in the left-hand panel. We determined that this Alternative qSOFA yielded generally reduced sensitivity and increased Time to (+) Screen, and it was therefore omitted from further investigation.

The qSOFA score was originally studied by Singer *et al*. in a population of patients who had blood cultures ordered and antibiotics initiated. As outlined above, the Sepsis-3 Task Force authors advised that meeting the qSOFA vital-signs criterion should “prompt consideration of possible infection in patients not previously recognized as infected”^7^. For the current investigation, we evaluated documented vital signs for each patient to determine the earliest time that the qSOFA vital-signs criterion was met, if at all. To do so, we used a comparable methodology as was used for evaluating the SPoT Sepsis vital-signs criterion. The qSOFA vital-signs criterion is fulfilled if at least two of the three constituent conditions are met: (1) Respiratory rate ≥ 22 per min; (2) GCS < 15; and (3) SBP ≤ 100 mmHg^7^. We determined the qSOFA vital-signs criterion to be positive upon documentation if any two of the three constituent conditions were met. We did *not* require the constituent vital-sign conditions to be documented within the same set of vital-signs: at any time that there was one documented vital-sign abnormality, we checked earlier in the record to see if there was prior documentation of a second vital-sign abnormality that would make the qSOFA vital-signs criterion positive; see Fig. 2. For all patients who met the qSOFA vital-signs criterion, we also determined their tCFI, as per the methodology described above. For comparison, we also determined which of these patients had antibiotics and blood cultures ordered (i.e., the methodology used by Singer *et al*.^7^ for determining suspected infection retrospectively – a criterion that cannot, of course, be used for prospective sepsis screening).

Through chart review, we aggregated additional subject characteristics, including age, triage vital signs, ED therapies, SOFA scores, and hospital mortality. Time to meeting vital-signs criteria (either SPoT or qSOFA) were determined. Study data were collected and managed using REDCap (Research Electronic Data Capture) data capture tools hosted at Partners Healthcare^13^. All clinical data extracted from paper charts (i.e., the derivation cohort) were corroborated by two separate chart reviewers, for accuracy.

### Analysis

For both cohorts, we aggregated characteristics for two subgroups: (1) those with below-average blood pressure at triage (SBP < 100 mmHg) for whom circulatory shock was potentially evident on arrival, and (2) those with triage SBP ≥ 100 mmHg who developed septic shock later in their ED stay. For the derivation cohort, we calculated the sensitivity of the sepsis screening rules and the times from triage to positive screens for these cohorts. For the validation cohort, we computed sensitivity, specificity, positive predictive value (PPV) and negative predictive value (NPV). We tested whether there were significant differences between the test characteristics of the screening rules, using the McNemar exact conditional test^14^ and the Fisher’s exact test, and between the times from triage to positive test using the Wilcoxon signed-rank test. Data distributions were reviewed for normality; a p-value < 0.05 was considered significant; only two-tailed tests were used. All analyses were done using STATA 14 (Stata, College Station, Texas) and R 3.4.3 (R Foundation, Vienna, Austria).

### Availability of Data and Material

The datasets used for this study are available from the corresponding author upon reasonable request.

## Results

### Derivation cohort

There were 141 patients in the derivation cohort who received vasopressors in the ED and were admitted to an ICU and treated for suspected sepsis. Of these, there were 71 (50%) who arrived at triage with SBP ≥ 100 mmHg; 95 (67%) presented with only “possible” infection (i.e., a non-specific symptom complex upon presentation). A Sepsis Risk Factor was present in 135 (96%) of ED septic shock patients. In terms of tCFI, 93 (66% of all ED septic shock patients) had at least one risk factor and “possible” infection; whereas 42 (30%) had a risk factor plus “probable” infection (Table 1).

**Table 1:** Clinical Characteristics of Septic Patients for Derivation Cohort & Validation Cohort.

	Parameter	Derivation Cohort Septic Patients (12-mo interval)	Validation Cohort Septic Patients (3-mo interval)
		(n = 141)	(n = 50)
Triage	Age, yrs, median (IQR)	70 (58–81)	65 (53–75)
	Febrile (Temp > 100.4 °F)	29 (21%)	5 (10%)
	Heart Rate, bpm, median (IQR)	104 (84–120)	97 (78–118)
	Systolic Blood Pressure, mmHg, median (IQR)	100 (76–120)	106 (87–135)
	Triage SBP < 100	70 (50%)	20 (40%)
	Triaged to Non-Acute ED Area	25 (18%)	3 (6%)
tCFI: triage Concern for Infection	In all patients:	n = 141	n = 50
	tCFI: Probable	46 (33%)	21 (42%)
	Possible	95 (67%)	28 (56%)
	Unlikely	0	1 (2%)
	In patients with a Sepsis Risk Factor:	135 (96%)	44 (88%)
	tCFI: Probable	42 (30%)	19 (38%)
	Possible	93 (66%)	24 (48%)
	Unlikely	0	1 (2%)
Clinical Details	Documented SBP < 90 in ED	135 (96%)	47 (94%)
	Time from Triage to SBP < 90, hours, median (IQR)	0.7 (0.0–2.5)	0.7 (0.1–2.1)
	Total IV Fluids, L, median (IQR)	3.00 (2.00–4.00)	2.65 (1.55–3.98)
	Serum Lactate, mmol/L, median (IQR)	2.7 (1.7–3.9)	2.6 (1.9–3.8)
	SOFA Score, median (IQR)	9 (7–11)	9 (7–12)
	Hospital Mortality	38 (27%)	15 (30%)
SPoT Vitals	Met SPoT Vital-Signs Criterion in the ED	141 (100%)	50 (100%)
	Time from Triage to SPoT, hours, median (IQR)	0.0 (0.0–0.4)	0.1 (0.0–0.8)
qSOFA Vitals	Met qSOFA Vital-Signs Criterion in the ED	127 (90%)	43 (86%)
	Time from Triage to qSOFA^a^, hours, median (IQR)	0.6 (0.1–1.8)	0.7 (0.1–1.4)

^a^Excludes patients who never met qSOFA vital-signs criterion in ED.

The ED sensitivities of the SPoT and qSOFA vital-signs criteria alone for vasopressor-dependent septic shock, without considering concern for infection, are reported in Table 1.

In addition, we computed sensitivities of the investigational criteria (i.e., vital-signs criteria *together with* tCFI as shown in Fig. 1) in the derivation cohort (not shown in Table 1). At triage, the sensitivity of SPoT-positive vital signs together with tCFI criterion was 71% (95% CI: 63–78%), while the sensitivity of qSOFA-positive vital signs together with tCFI criterion was 33% (95% CI: 26–42%), which were significantly different, p < 0.001. Overall sensitivities (i.e., at triage *or* any time later in the ED) were also significantly different: 99% (95% CI: 96–100%) and 89% (95% CI: 82–93%), respectively, p < 0.001. The associated median times from triage to meeting these investigational criteria were 0.0 hours (IQR: 0.0–0.4 hours) and 0.6 hours (IQR: 0.1–1.8 hours), respectively, which were significantly different, p < 0.001. For qSOFA, we did not require the constituent vital-sign conditions to be documented within the same set of vital-signs, and in 21% of the cases, it was necessary to rely on SBP, RR, or GCS that were documented at different times.

Because the derivation cohort only consisted of patients with septic shock, it was not possible to evaluate other diagnostic test characteristics, e.g., specificity or positive predictive value.

### Validation cohort

There were 19,670 total adult ED patient visits during the 3 months studied. Of these, 2,528 met the SPoT and/or the qSOFA vital-signs criteria. The associated charts were reviewed to adjudicate whether the patients had “possible” or “probable” bacterial infection and a sepsis risk factor upon initial ED evaluation. From these adjudications, it was possible to evaluate tCFI. For the 1,701 charts (67% of the total) that were reviewed by two team members, Cohen’s Kappa for adjudicating whether there was “possible” or “probable” infection was 0.80; for adjudicating whether risk factors were present, Cohen’s Kappa was 0.91.

There were 50 patients who developed vasopressor-dependent septic shock. Table 2 presents clinical details of the 50 septic shock patients grouped by clinician’s initial, pre-diagnostic judgment of infection: “probable”, “possible”, or “unlikely”. Of these 50 patients, 30 (60%) presented at triage with SBP ≥ 100 mmHg; 28 (56%) presented with only “possible” infection (i.e., a non-specific symptom complex upon presentation). Additional characteristics of this cohort are also reported in Table 1. In terms of tCFI, 24 (48%) ED septic shock patients had a sepsis risk factor and “possible” infection upon presentation; whereas 19 (38%) had a risk factor plus “probable” infection.

**Table 2:** Clinical Summary of Septic Patients for Validation Cohort.

Age (yrs)	Major Comorbidities	Systemic Sx	Localizing Sx	tCFI	Triage SBP (mmHg)	Triage HR (bpm)	Triage Temp (°F)	ICU Dx
**“Likely” Pre-diagnostic Infection Probability**								
27	IBD, TPN	Fever, weakness	“Generalized” abd. pain, HA	+	104	131	101.7	Chole
27	*Unknown*	Unresponsive	*Unknown*	+	70	88	96.4	Pna
31	Onc	Fever	SOB	+	98	160	105.1	Pna
44	Onc	Fever, hypotension	+/− abd. pain	+	100	137	97.8	Uro
46	ESRD, C. Dif.	Weakness	Diarrhea	+	146	68	98.4	Colitis
51	ESLD, auto	“Chronic body pain”	Nausea, cough, SOB	+	95	80	97.0	Colitis vs pna
53	Major disability	Unresponsive	*Unknown*	+	90	97	98.7	Uro
54	Onc	Weakness	Difficulty swallowing, cough	+	180	113	98.5	Pna
55	ESLD	Somnolent	Leg pain, bilateral leg edema	+	108	65	97.0	Soft tissue
58	Onc	Fever, tachycardia	None	+	124	159	104.0	Uro vs chole
59	Major disability	Fever	SOB	+	125	122	97.3	Uro
65	Major disability	Fever	Dry mouth, shoulder pain	+	123	88	100.2	Pna vs uro
65	COPD, CHF, C. Dif.	Lethargy	Abd. pain, diarrhea	+	88	110	97.0	Uro
66	*Unknown*	*Unknown*	SOB	+	149	120	96.0	Pna
66	Major disability	Fever, lethargy	SOB	+	160	112	98.4	Pna vs uro
67	*Unknown*	Unresponsive	SOB	+	73	82	98.0	Pna & endo
71	None	Fever	Acute femur fx after fall	+	101	83	98.0	Uro
81	COPD, CAD	Confusion	Abd. pain, SOB	+	101	79	98.2	Peritonitis
85	Major disability	Fever, lethargy	*Unknown*	+	130	103	100.1	Uro
89	COPD	Respiratory arrest	*Unknown*	+	87	114	94.6	Pna
94	CHF	Fever	Cough	+	120	70	97.2	Pna
**“Possible” Pre-diagnostic Infection Probability**								
27	None	Near-syncope	Breast pain & erythema, SOB	*Neg*	87	144	99.9	Mastitis
38	ESLD	Lethargy, confusion	“Mild” abd. pain	+	190	69	98.0	Chole
43	None (alcoholism)	Somnolent, confused	MODS (from OSH)	*Neg*	133	109	96.5	Empiric
45	ESLD	Active seizures	Hypoxia	+	163	144	101.7	Pna
48	Onc	None	Abd. pain, vomiting, SOB	+	73	72	98.2	Peritonitis
50	IBD, TPN	Weakness, falls	Abd. pain	+	98	95	98.4	Line
53	Active cancer, COPD	Weakness	Chest & back pain	+	143	107	100.0	Empiric
54	Major disability	Fever, confusion	Orthopedic cast (foot) pain	+	109	118	102.5	Uro
56	None (minor CVA)	Confusion, syncope	Chest pain, SOB	*Neg*	159	81	97.0	Empiric
56	ESLD	Weakness	Chest pain	+	58	86	97.1	Uro & colitis
57	Onc	Lethargy, hypotension	Hemoptysis	+	97	116	97.4	Empiric
59	CHF	*Unknown*	SOB	+	126	138	97.0	Pna
60	None (alcoholism)	Hypotensive	Abd. & chest pain, EKG Δ	*Neg*	74	122	98.0	Odontogenic
64	ESLD	Somnolent, confused	*Unknown*	+	96	96	97.9	Colitis vs uro
67	None	Somnolent, confused	*Unknown*	+	77	60	97.0	Empiric
67	None	Lethargy, syncope	*Unknown*	+	103	68	86.3	Empiric
69	ESLD	Somnolent, confused	*Unknown*	+	67	73	97.8	Soft tissue
70	None	Lethargy, confusion	Incontinence	+	148	110	99.4	Soft tissue
72	COPD	Lethargy, diaphoretic	*Unknown*	+	64	42	98.0	Empiric
74	CHF	None	Back pain, cough, SOB	+	126	80	97.8	Pna
75	Onc	None	“Mild” RUQ pain, nausea	+	186	89	96.0	Chole
75	None	Syncope	Nausea	+	78	130	97.4	Uro
77	Onc	Myalgias	HA, SOB	+	79	71	97.0	Line
79	*Unknown*	PEA arrest	*Unknown*	+	129	116	97.5	Pna
79	Major disability	Somnolent, confused	*Unknown*	+	137	105	98.2	Uro
80	Onc	None	“Diffuse” abd. pain, SOB	+	159	128	97.0	Pna
81	Appi (recent)	Syncope	Diarrhea, SOB	+	136	65	97.1	Peritonitis
84	CHF, COPD	Somnolent	SOB	+	111	77	96.2	Pna
**“Unlikely” Initial Infection Probability**								
82	None	“Dizziness”	Significant bradycardia	*Neg*	128	44	98.0	Urosepsis

Clinical summary of vasopressor-dependent ED septic shock patients from validation cohort. “Unknown” indicates clinical information that ED providers were unable to obtain, e.g., patient confused or mechanically ventilated. *Initial infection probability*: Categorization of ED provider concern for bacterial infection prior to diagnostic testing (“likely”, “possible” or “unlikely”) based on the ED provider’s documentation. *Major comorbidities*: Pre-specified sepsis risk factors (see text for details); notable PMHx that was not included in the tCFI “risk factor” criteria is reported in parentheses. Onc = active cancer; major disability = physical or cognitive disability requiring services; COPD = chronic obstructive pulmonary disease; CAD = coronary artery disease; ESLD = end-stage liver disease including cirrhosis; auto = auto-immune disease with immunosuppressive therapy; CHF = congestive heart failure; C. Dif. = recent or active treatment for C. Dif. colitis; h/o CVA = prior cerebrovascular accident; appi (recent) = recent appendicitis with ongoing antibiotic treatment. *Systemic sx* and *Localizing sx*: pertinent positives documented in ED provider note. Sx = symptoms; abd. = abdominal; +/− = ED documentation inconsistent; HA = headache; SOB = shortness of breath; fx = fracture; EKG Δ = ST segment elevations on electrocardiogram; RUQ = right upper quadrant of abdomen; MODS (from OSH) = transferred from outside hospital with multiple organ failure. *ICU Dx*: documented indication for antibiotic treatment following admission to the ICU. Chole = cholesystitis; uro = urosepsis; pna = pneumosepsis; empiric = antibiotics without identified source; line = indwelling vascular catheter infection.

Unlike the derivation cohort, the validation cohort consists of all ED patients (i.e., with and without the investigational outcomes), allowing for the calculation of diagnostic test characteristics. For the SPoT and qSOFA vital-signs criteria (without consideration of tCFI) diagnostic test characteristics are reported in Table 3 (top section).

**Table 3:** Test Characteristics of SPoT and qSOFA Vital-Signs Criteria for Validation Cohort.

	1° Outcome: ED Vasopressors, Direct ICU Admission, and Treatment for Infection					2° Outcome: ICU Admission Within 24 hrs and Treatment for Infection		
	Triage Sensitivity	Time to (+) Screen	Sensitivity^a^	Specificity^a^	Positive Predictive Value^a^	Sensitivity^a^	Specificity^a^	Positive Predictive Value^a^
**I. Diagnostic Test Characteristics of Vital-Signs Criterion Alone**								
SPoT Vitals	60% (45–74%)^ǂ^	0.1 hours (0.0–0.8)^*^	100% (93–100%)^*^	89.1% (88.7–89.6%)^¶^	2.3% (1.7–3.0%)^*^	73% (67–79%)	89.6% (89.1–90.0%)^¶^	7.1% (6.1–8.3%)^¶^
	30/50		50/50	17,488/19,620	50/2,182	155/212	17,431/19,458	155/2,182
qSOFA Vitals	28% (16–43%)^ǂ^	0.7 hours (0.1–1.4)^*^	86% (73–94%)^*^	94.2% (93.8–94.5%)^¶^	3.6% (2.6–4.9%)^*^	69% (62–75%)	94.7% (94.4–95.0%)^¶^	12.4% (10.5–14.4%)^¶^
	14/50		43/50	18,481/19,620	43/1,182	146/212	18,422/19,458	146/1,182
**II. Diagnostic Test Characteristics of Vital-Signs Criterion together with tCFI Criterion**								
SPoT Vitals	56% (41–70%)^ǂ^	0.1 hours (0.0–0.8)*	90% (78–97%)	95.3% (95.0–95.6%)^¶^	4.7% (3.4–6.2%)	63% (56–70%)	95.7% (95.5–96.0%)^¶^	13.9% (11.8–16.3%)^¶^
	28/50		45/50	18,704/19,620	45/961	134/212	18,631/19,458	134/961
qSOFA Vitals	28% (16–43%)^ǂ^	0.7 hours (0.0–1.5)^*^	80% (66–90%)	97.0% (96.7–97.2%)^¶^	6.3% (4.5–8.5%)	60% (53–67%)	97.4% (97.2–97.6%)^¶^	20.0% (16.9–23.3%)^¶^
	14/50		40/50	19,024/19,620	40/636	127/212	18,949/19,458	127/636
**III. Diagnostic Test Characteristics of Vital-Signs Criterion together with Singer’s “Suspected Infection” Criteria** (**Antibiotics and Blood Cultures Ordered**)^**b**^								
SPoT Vitals	50% (36–64%)^ǂ^	N/A^b^	80% (66–90%)	98.5% (98.3–98.7%)^¶^	12.0% (8.7–16.0%)	52% (45–59%)	98.9% (98.7–99.0%)^¶^	33.3% (28.3–38.7%)
	25/50		40/50	19,327/19,620	40/333	111/212	19,236/19,458	111/333
qSOFA Vitals	24% (13–38%)^ǂ^	N/A^b^	70% (55–82%)	98.9% (98.7–99.0%)^¶^	14.0% (9.9–18.9%)	51% (44–57%)	99.3% (99.1–99.4%)^¶^	42.8% (36.6–49.2%)
	12/50		35/50	19,405/19,620	35/250	107/212	19,315/19,458	107/250

Pairwise statistical comparisons of results for SPoT and qSOFA vital-signs criteria. For proportions, the percentages (and 95% confidence intervals) are shown above, as well as the raw counts for the relevant numerator and denominator in terms of ED subjects from the validation cohort. For times, the median (and interquartile range) are shown. For statistical tests-of-significance, we applied the McNemar exact conditional test for comparing sensitivities and specificities; the Fisher’s Exact Test for comparing positive predicative value; and the Wilcoxon signed-rank test for scalar results.

tCFI: triage criteria for infection (see Methods Section and Fig. 1 for details).

^a^Based on patients meeting vital-signs criteria *at any time* during ED course.

^b^Antibiotics & blood cultures are retrospective criteria for suspicion for infection as per Singer *et al*.^7^ which cannot be applied for prospective sepsis screening/decision-making.

*Significant difference, p < 0.05, in pairwise comparison between SPoT Vitals and qSOFA vitals, within each section (I, II and III) above.

^ǂ^Significant difference, p < 0.01, in pairwise comparison between SPoT Vitals and qSOFA vitals, within each section (I, II and III) above.

^¶^Significant difference, p < 0.001, in pairwise comparison between SPoT Vitals and qSOFA vitals, within each section (I, II and III) above.

Table 3 (middle section) reports complete diagnostic test characteristics for vital-signs criteria used in conjunction with tCFI. At triage, the sensitivity of SPoT-positive vital signs together with tCFI criterion was 56% (95% CI: 41–70%), while the sensitivity of qSOFA-positive vital signs together with tCFI criterion was 28% (95% CI: 16–43%), which were significantly different, p < 0.01. Overall sensitivities (i.e., at triage *or* any time later in the ED) were 90% (95% CI: 78–97%) and 80% (95% CI: 66–90%), respectively, which were not significantly different, p > 0.05. The associated median times from triage to meeting the investigational criteria (vital-sign criterion plus tCFI criterion) were 0.1 hours (IQR: 0.0–0.8 hours) and 0.7 hours (IQR: 0.0–1.5 hours), respectively, which were significantly different, p < 0.05. For qSOFA, we did not require the constituent vital-sign conditions to be documented within the same set of vital-signs, and in 14% of the cases, it was necessary to rely on SBP, RR, or GCS that were documented at different times.

As a basis for comparing the aforementioned results, Table 3 (bottom section) shows diagnostic test characteristics of the vital-signs criteria with the Singer retrospective criteria for suspected infection (i.e., ordering antibiotics and blood cultures in the ED). Diagnostic test characteristics for the secondary outcome are also shown in Table 3.

## Discussion

We can consider the implications of our findings in terms of (a) the use of vital signs for sepsis screening, (b) how to determine possible infection in ED patients not known to be infected, and (c) the overall feasibility of ED sepsis screening prior to diagnostic testing. Regarding the use of vital signs, we found that the SPoT vital-signs criterion, using just SBP and HR, can achieve significantly higher sensitivity at triage compared with the qSOFA score, 56% versus 28%, respectively, in patients who also met triage concern-for-infection (tCFI) criterion. When applied to vital signs subsequently measured throughout the patients’ ED stays, both rules ultimately identified most of the septic shock patients (90% and 80% in the validation cohort, respectively), with the SPoT Sepsis rule identifying patients significantly earlier in their ED stays (p = 0.03), albeit with a small but statistically significant reduction in specificity (p < 0.001).

The reduced sensitivity of the qSOFA rule compared to the SPoT vital-signs criterion may be – in part – because GCS and RR can be problematic to measure accurately: identification of confusion (e.g., GCS < 15) requires a dialogue with the patient, while the RR measurement is notably problematic^15^. The SPoT rule has a procedural advantage in that SBP and HR are essentially always obtained at the same time and can be assessed and re-assessed simply by glancing at the patient’s monitor. Also, another subtlety regarding the qSOFA score is that the associated vital-signs abnormalities were sometimes documented at *asynchronous* times. Operationally, this means that when one abnormal vital sign is documented, caregivers also need to be aware of prior abnormalities to successfully identify patients at risk for ED sepsis using the qSOFA score.

Regarding how to determine possible infection in ED patients not known to be infected, our findings suggest that septic shock patients commonly presented with non-specific symptoms. Prior to diagnostic testing, bacterial infection was only a diagnostic *possibility*, but most often not probable, for most cases. Presenting complaints were often non-specific for bacterial infection, such as isolated fever without localizing signs, weakness, or non-localizing abdominal pain. The clear implication is that a screening tool for early detection of sepsis must anticipate that many ED patients will have non-specific symptoms.

The term “suspected infection”^4,16^, which is used in the sepsis literature, risks interpretation by practicing clinicians as referring to a patient with *probable* bacterial infection. Our data suggest that this may represent the minority of septic shock patients upon initial presentation. This is not merely a matter of semantics if there are delays in evaluating and testing in the ED because sepsis is not “suspected” until after the fact (i.e., after the return of confirmatory diagnostic data). One possible advantage of the tCFI criterion is that it explicitly encourages clinicians to consider sepsis in patients with non-specific symptoms.

Of course, positive vital-signs criteria applied to all patients who present to triage will lead to many false alarms. To mitigate this, we introduced the concept of tCFI, which combines “possible infection” with a “sepsis risk factor” (either age or a major comorbidity or *in extremis* state). Applying tCFI criterion to the vital-signs rules doubled the rate of true-positives without substantial loss of sensitivity (Table 3 – middle section).

The justification for focusing on patients with risk factors was that 96% of ED patients admitted to the ICU with sepsis in our derivation cohort had at least one sepsis risk factor (Table 1). There was also a minority of previously healthy, younger patients with sepsis and without risk factors (4% and 12% in the derivation and validation cohorts, respectively). However, a proportion of these were deemed tCFI-positive by having high likelihood of bacterial infection (“probable” infection) prior to diagnostic testing (Fig. 1).

Our findings also suggest that ED clinicians might underestimate the likelihood of serious infection. Only 80% of septic shock patients in the validation cohort that met our primary outcome had “suspected infection” according to the Sepsis Definitions Task Force criteria (i.e., ED blood cultures and antibiotics ordered). Moreover, there were septic shock patients who did not meet the tCFI criterion, based on our retrospective review of the treating clinician’s documented information (Table 3). The specifics of two of these cases may serve to illustrate how ED clinicians can underestimate the likelihood of infection: (1) a patient with polysubstance abuse, ST-elevations and hypotension who went to the cardiac catheterization lab on vasopressors before acute coronary syndrome was ruled-out and the patient was subsequently diagnosed with sepsis and bacteremia caused by an oral infection; and (2) a geriatric patient admitted to the cardiac ICU on vasopressors thought primarily due to bradycardia who was subsequently found to have urosepsis. Anecdotally, these cases suggest that ED clinicians can underestimate which patients have “possible” or “probable” bacterial infection, which only reinforces the need for a sepsis screening rule to accommodate patients with non-specific presenting symptoms.

Taken altogether, the findings of this analysis affirm the feasibility of sepsis screening at triage. Most septic shock patients could be identified upon triage, or shortly thereafter, using only vital signs and the patient’s risk factors and symptoms. Such patients can and should be prioritized for rapid evaluation and diagnostic testing to confirm infection and initiate treatment expeditiously. Our departmental guidelines advise that an ED provider and ED nurse should promptly evaluate patients who screen positive for possible sepsis either at or shortly after triage and, within an hour or less, obtain sufficient diagnostic data to decide whether to administer fluids and initiate antibiotics. The potential importance of a high-sensitivity triage sepsis screen is apparent considering that most septic shock patients had non-specific symptoms, were afebrile and had SBP ≥ 100 mmHg at triage. Without a high-sensitivity screening protocol in place such innocuous presentations may lead to delays in evaluation, diagnostic testing, and initiation of antibiotics.

The PPVs of the sepsis screening rules were modest, 4.7% and 6.3% for the SPoT Sepsis rule and qSOFA, respectively. However, the expected clinical response is not resource intensive (only requiring evaluation by one provider and one nurse). This seems reasonable, noting that entire trauma teams are activated for trauma populations with <10% incidence of clinically significant hemorrhage^17^. Likewise, stroke teams are activated for <10% likelihood of patients qualifying for reperfusion therapy^18,19^. The PPV for our secondary outcome – admission to an ICU within 24 hours with treatment for suspected infection – was higher (13.9% and 20.0% for SPoT and qSOFA, respectively), suggesting that these criteria do identify high-acuity ED patients worthy of expedited evaluation. In the future, high-sensitivity screening rules might also be used to identify patients who require rapid biomarker testing, if reliable and high-specificity point-of-care diagnostic tests for sepsis become available.

There are several limitations to this report. First, it relied only on documented, retrospective data. Clinician documentation is not always reliable. Also, it is possible that the findings might change given increased clinician awareness of the value of sepsis screening and/or additional training about how to reliably identify GCS < 15 and measure RR accurately. Second, the SPoT Sepsis rule’s findings have only been validated in a single medical center, and the generalizability of the rule to other EDs remains an open question. The qSOFA score, by contrast, has been evaluated in very large databases as a predictor of mortality in patients treated for suspected bacterial infection^7^.

Finally, and perhaps most importantly, the actual clinical pay-off for a sepsis screening rule, such as the SPoT Sepsis rule or qSOFA, remains unknown. Recent reports suggest there is a reduction in survival associated with every hour of antibiotic delay^4,20^, but this has not been verified through prospective evaluation. Even less is known about whether sepsis can even be aborted altogether if therapy is provided in the hours just before its onset (e.g., in most septic patients who present to the ED with triage SBP ≥ 100 mmHg). Finally, little is known about whether there are specific patient subpopulations (e.g., patients with immunosuppression or major comorbidities) who are uniquely sensitive and vulnerable to delayed interventions such as antibiotics; if so, it would be important to develop screening methods for any specific, time-sensitive sub-population. The SPoT Sepsis rule focuses earlier attention on patients with major comorbidities, but whether this is clinically useful must be further investigated.

Both the SPoT Sepsis rule and qSOFA score were sensitive indicators in ED patients with sepsis requiring ICU admission. The SPoT Sepsis rule was more likely to be positive at triage, and was positive significantly earlier, which may be advantageous if the goal is to screen for sepsis at triage or early in the ED stay. Of note, many of the ED septic shock patients presented with non-specific complaints, which must be accounted for by any screening rule intended to make sepsis care analogous to other high-acuity conditions, in which high-risk, undifferentiated patients are identified at triage for priority evaluation.
